# Modulation of Biofunctional and Structural Properties in Stevia‐Sweetened Ice Cream by Lactic Fermentation

**DOI:** 10.1002/fsn3.72030

**Published:** 2026-06-14

**Authors:** Tulay Ozcan, Gokce Keser, Ali Riza Kaya, Yunus Emre Cetin, Fatime Elciboga

**Affiliations:** ^1^ Department of Food Engineering Bursa Uludag University Bursa Turkiye; ^2^ Inventa Food Company Bursa Turkiye

**Keywords:** cocoa, dietetic, ice cream, postbiotic system, probiotic

## Abstract

This study investigated the viability of probiotic *Bifidobacterium animalis* subsp. *lactis* and *Lactobacillus gasseri* in a dietetic ice cream matrix. Four formulations were produced: control (A), *B. animalis* subsp. *lactis* (B), *L. gasseri* (C), and a coculture of both strains (D). A synbiotic frozen system was developed using a milk‐based cocoa matrix, and the cold adaptation of probiotic cells was monitored in the presence of phenolic‐stevia‐based cryoprotective components. Samples were analyzed before freezing, after freezing, and during 60 days of storage. Bacterial viability was assessed in the synbiotic ice cream matrix together with physicochemical properties (pH, texture, color, and organic acid formation), phenolic, antioxidant capacity (CUPRAC, DPPH), and sensory characteristics. Although a decrease in viable cell counts was observed during storage, levels remained above the recommended minimum threshold (≥ 6 log_10_ cfu g^−1^), indicating the maintenance of probiotic functionality. Microbial counts were positively correlated with lactic acid production, textural parameters (penetration and adhesiveness), and sensory attributes, such as sourness and fermented milk flavor. The synbiotic fermented system exhibited higher antioxidant capacity. Sensory evaluation showed that all fermented ice cream samples achieved acceptable scores (> 4 on the hedonic scale), indicating good consumer acceptability throughout storage. Overall, the findings suggest that the stevia‐sweetened cocoa ice cream matrix can serve as a promising synbiotic frozen carrier for maintaining probiotic viability during 60 days of storage, while retaining acceptable functional and sensory quality.

## Graphical abstract

This study investigated the viability of probiotic *Bifidobacterium animalis* subsp. *lactis* and *Lactobacillus gasseri* in a dietetic ice cream matrix. Overall, the findings suggest that the stevia‐sweetened cocoa ice cream matrix can serve as a promising synbiotic frozen carrier for maintaining probiotic viability, while retaining acceptable functional and sensory quality.

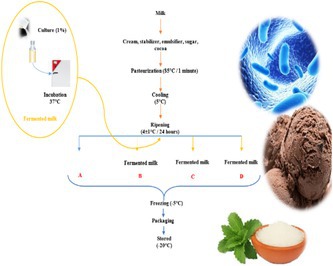

## 1 Introduction

Functional foods contain additional components to provide a specific health benefit and the nutritional effects they create with their natural content. Among these foods, products containing probiotic bacteria and modulating the intestinal microbiota have an important place (Birch and Bonwick 2019; John 2021).

The combined application of prebiotics or probiotics aims to affect the intestinal environment, where trillions of bacteria live, to benefit the organism's health. In synbiotic systems, the most crucial activity of prebiotics is to impact intestinal microflora by increasing the number of activities of beneficial bacteria. This can lead to a decrease in the population of potentially pathogenic microorganisms or the prevention of harmful metabolic activities in the host microbiota (Saad et al. 2013; Mohanty et al. 2018; Keser and Ozcan 2025). On the other hand, probiotic strains produce health effects through one or more metabolic pathways from several defined mechanisms. Probiotics impact the intestinal ecosystem by affecting mucosal immune mechanisms, interacting with pathogenic bacteria, producing metabolic products such as short‐chain fatty acids (SCFAs) and linking with host cells through chemical signaling. These mechanisms lead to the antagonism of potential pathogens, an improved gut environment, strengthening of the gut barrier, regulation of inflammation, and increased regulation of the immune reaction to antigenic challenges (Sims et al. 2014; Bindels et al. 2015; Rinninella and Costantini 2022).

Prebiotics are generally used as growth/development substrates for bacteria or are selectively or specifically accessible to microorganisms in the colon because they are nutritional components of microorganisms. In general, the ability of prebiotics to act as substrates and to be fermented may vary depending on the prebiotic and the strain, affecting bacterial metabolism and vitality. Many studies have determined the prebiotic potential of various plant extracts and phenolic compounds (de Vrese and Schrezenmeir 2008; Figueroa‐González et al. 2011; Holmes et al. 2020). Prebiotics, which are indigestible dietary carbohydrates, are fermented by colon bacteria and modulate the intestinal microbiota by providing the formation of SCFAs such as acetate, propionate, and butyrate (Shokryazdan et al. 2017; Neri‐Numa et al. 2020; Pujari and Banerjee 2021).

Since probiotics are live, they are prone to inactivation during product storage. Manufacturers usually produce products containing high bacterial counts so that the product does not fall below the potency declared on the label at the end of its shelf life. Probiotic bacteria must resist environmental stress during production and shelf life (Gueimonde and Sanchez 2012; Hill et al. 2014; Karaman and Ozcan 2021).

Ice cream is a dairy product with sensory accepted worldwide recognition, and potential for commercial growth due to its numerous flavor options and combinations. The addition of probiotic cultures and prebiotics transforms ice cream into a potential nutraceutical food with the formation of health‐promoting components (Cruz et al. 2009; de Souza et al. 2010; Ferraz et al. 2012; Goff and Hartel 2013; Balthazar et al. 2015).

There are many types of foods with different nutritional compositions on the market. Nutritional compositions and functional effects should be created for specific populations or consumer groups such as young, old, athletes. It is essential to provide multifaceted and nutritious diets to growing children (e.g., infants and young children) to help them meet their daily micro and macronutrient requirements. In addition, consuming healthy, nutraceutical, safe, and reliable foods also has a unique value for future generations (Gueimonde and Sanchez 2012; Verduci et al. 2021). With a sustainable approach, consumers' association of high‐sugar foods with obesity, diabetes, heart disease, and high blood pressure increases the tendency toward reduced‐sugar products in new‐generation nutritional models. The sensory experience of sucrose, along with the senses such as flavor and taste that consumers experience with naturally sweetened probiotic health products, continues to be a driving force for purchase (Silveira et al. 2023; Crown et al. 2024). The novelty of this study lies in providing, to the best of our knowledge, the first comparative assessment of *Bifidobacterium animalis* subsp. *lactis* and *Lactobacillus gasseri* as probiotic cultures in a diet ice cream matrix. This study aimed to investigate the viability of probiotic *B. animalis* subsp. *lactis* and *L. gasseri* strains in stevia‐fortified cocoa ice cream by identifying textural and sensory correlations.

## 2 Materials and Methods

### 2.1 Material

Milk (0.5 g/100 g fat), milk cream (18 g/100 g fat), stevia (total steviol glycoside ≥ 95.0, PureCircle Sdn Bhd, Malaysia), skim milk powder and stabilizer mixture (CMC, carob gum, xanthan gum, pectin) (0.5 g/100 g) obtained from local companies in Turkiye. Vanilla and cocoa were purchased from Pakmaya (Izmit, Turkiye). In the study, the culture containing *Bifidobacterium animalis* subsp. *lactis* (HN019) bacteria were obtained from DuPont️ Danisco (Copenhagen, Denmark). *Lactobacillus gasseri* was obtained from SFA RandD Company (Istanbul, Turkiye).

### 2.2 Method

#### 2.2.1 Culture Preparation

Probiotic strains ( *B. animalis* subsp. *lactis* and *L. gasseri* ) were activated in MRS broth medium, a second multiplication was made in the milk system (3 g/100 g), and when they reached ~9 log_10_ cfu/g in the fermented milk system.

#### 2.2.2 Preparation of Probiotic Ice Cream Matrix

**FIGURE 1:**
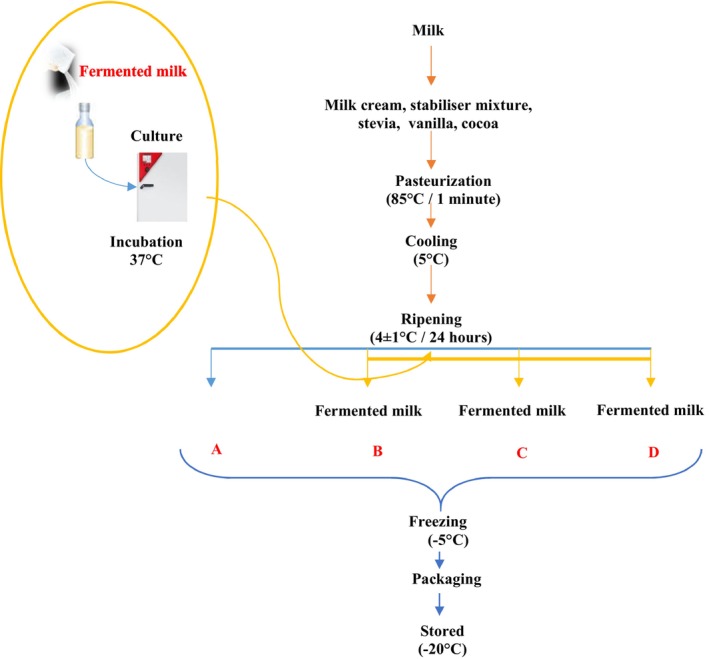
Probiotic ice cream production. (A) Control ice cream; (B) ice cream containing *Bifidobacterium animalis* subsp. *lactis*; (C) ice cream containing *Lactobacillus gasseri* ; (D) ice cream containing *Bifidobacterium animalis* subsp. *lactis* + *Lactobacillus gasseri*.

Ice cream production was carried out according to the method described by Salem et al. (2005) and Ozcan‐Yilsay et al. (2006). Ice cream mixes were produced according to 32.87 g/100 g dry matter, containing milk, milk cream, skimmed milk powder, stevia, vanilla, cocoa and stabilizer mixture. Ice cream mixes were heated at 85°C for 1 min, and all mixes were cooled to 5°C and matured at the same temperature for 24 h. Fermented culture milk systems were replaced (50 g/100 g milk replacement) to the aged mixes at 37°C in probiotic ice creams (B, C, D) before freezing. After the batch freezing system, ice cream samples were filled into 100 mL plastic containers. The frozen probiotic milk matrix was stored at −18°C, and therapeutic and technological quality properties were determined by microbiological, physicochemical, and sensory analyses during the 60‐day storage period. The production flow chart is presented in Figure 1. The experimental groups were defined as follows: A, control ice cream (none strains), B, ice cream inoculated with *B. animalis* subsp. *lactis*; C, ice cream inoculated with *L. gasseri* ; and D, ice cream inoculated with *B. animalis* subsp. *lactis* + *L. gasseri* . The ice cream formulations were produced in two independent batches, and all analyses were conducted in triplicate for each batch.

#### 2.2.3 Microbiological Analysis

The counts *B. animalis* subsp. *lactis* and *L. gasseri* were determined directly in the mix (before freezing), after freezing the mix, and storage time of the ice cream samples. The viable cell numbers were counted out using the pour‐plate technique. The counts of *B. animalis* subsp. *lactis* were determined in MRS (Man, Rogosa and Sharpe) (Merck, Germany) agar containing LiCl (0.2 g/100 mL) and C_3_H_5_NaO_2_ (0.3 g/100 mL). *L. gasseri* was enumerated on MRS agar. Petri dishes were incubated with AnaeroGen (Oxoid, Basingstoke, England) at 37°C for 72 h. The colonies formed in the petri dishes were counted, and the microorganism viability (Viability Index) was calculated according to the equation (Karaman and Ozcan 2021; Ozcan et al. 2021):

$$\begin{array}{c} \mathrm{VI}=\text{Final cell population}\;\left(\log_{10}\;\mathrm{cfu}/\mathrm{mL}\right)/\\ \;\text{Initial cell population}\;\left(\log_{10}\;\mathrm{cfu}/\mathrm{mL}\right) \end{array}$$

#### 2.2.4 Physicochemical Analysis

The pH values were determined by a portable pH meter (Wtw pH 3110 Set 2, Germany). The samples' L*, a*, and b* values were determined with Minolta Chromameter CR‐400 (Konica Minolta Co. Ltd., Osaka, Japonya).

The BI* values were calculated with the equation (Al‐Hilphy et al. 2022):

$${\mathrm{BI}}^{*}=\left[100\;\left(X-0.31\right)\right]\;0.172\;X=\left(a^{*}+1.75L^{*}\right)\;\left(5.645L^{*}+a^{*}-3.012b^{*}\right)$$

The textural properties were determined with a Texture Analyzer (TA.XT plus, Stable Micro Systems Ltd., UK) with a cylinder probe (P/5). The samples were compressed at a speed of 10 mm/s at −10°C ± 1°C temperature. The penetration depth was 12 mm, and the probe speed was 2.0 mm/s. The obtained curves were used to determine firmness (maximum penetration) (g), work of penetration (shows of sample softness) (g·s), and resistance to probe withdrawal (adhesiveness) (g·s) to separate the probe from the samples (Pon et al. 2015; Lis et al. 2021).

Extraction of organic acids was carried out by modifying the method determined by Vénica et al. (2014). 5 g of sample was weighed and diluted to 50 mL with 10 nM H_2_SO_4_, vortexed for 1 min, then centrifuged at 4000 rpm for 20 min. The upper phase was passed through a 0.45 μm PVDF filter and transferred to HPLC vials. The Shimadzu LC‐2030C 3D Plus model HPLC system with a PDA detector (190–800 nm) was used in organic acid analysis. Chromatographic separation, Thermo Scientific AcclaimTM, Organic Acid, 120 Å, C18 (4.0 × 250 mm, 5 μm) analytical column, 100 mM Na_2_SO_4_ (0.6 mL/min flow rate, column temperature 30°C, injection volume 10 μL) was used as the mobile phase. Organic acid peaks were determined at 210 nm wavelength as lactic and acetic acid.

The total phenolic compound (TPC) in ice cream samples was determined using the Folin–Ciocalteu method (Lee et al. 2009; Dogan et al. 2021). DPPH (2.2‐diphenyl‐1‐picrylhydrazyl) and CUPRAC (cupric ion‐reducing) determined the total antioxidant activity of ice cream samples. 0.25 mL of extract, 2.5 mL of stock DPPH and 2.5 mL of methanol were added and kept in the dark for 1 h; then absorbance values were recorded at 517 nm. Absorbance values were calculated as mg TE 100^−1^ with the formula created from the Trolox curve (Ozcan et al. 2019; Dogan et al. 2021). The CUPRAC assay was performed according to Boyanova et al. (Boyanova et al. 2022). The total antioxidant capacity of the samples was determined using a Trolox standard curve, and the results were expressed as a sample of mg Trolox equivalent TE 100 g^−1^.

#### 2.2.5 Sensory Analysis

Sensory evaluation of the samples was conducted in accordance with ISO 4121: 2003 in the sensory analysis laboratory (International Organization for Standardization 2003). The panelists evaluated consumer acceptability with a hedonic scale (1–5) (5‐Liked it very much, 4‐Liked it, 3‐Neither liked it nor disliked it, medium, 2‐Did not like it, 1‐Did not like it at all). The samples were also evaluated for consumer purchase intentions using an evaluation form containing the scoring process: “1 = Definitely would not buy, 2 = Probably would not buy, 3 = May or may not buy, 4 = Probably would buy, 5 = Definitely would buy” (Mora et al. 2022).

Quantitative Descriptive Analysis (QDA) was performed on four probiotic ice cream samples. QDA was performed by a trained sensory panel according to the general guidance for establishing a sensory profile described in ISO 13299: 2016. Nine trained panelists (certified in Technical Competence and Validation in Sensory Analysis of Foods, seven women and two men aged 20–55) evaluated the intensities of the flavor and texture characteristics of the samples (International Organization for Standardization 2016). The panelists were trained in the descriptive sensory analysis of probiotic foods, and the use of a trained panel was preferred to ensure consistent and repeatable evaluation of the flavor and texture characteristics of the samples under controlled laboratory conditions. The same panel was used for both hedonic and descriptive analysis. Panelists were trained in descriptive sensory analysis of probiotic foods, and informed consent was obtained. A well‐established dictionary was used with a universal intensity scale of 0–15 points consistent with the spectrum descriptive analysis method. (0 = not perceived; 7–8 = moderate intensity/close to reference standard; 15 = extremely intense). Each ice cream sample was served in 100 mL capped containers with a random 3‐digit code at −15°C to −10°C. Twice, each panelist in a separate panel evaluated each sample (Crown et al. 2024).

In this study, participants were informed about the product content and the relevant principles and guidelines for the sensory evaluation of ice cream samples. This study involved sensory analysis conducted by a trained panel. Although there was no formal ethics committee, informed consent was obtained from all participants to ensure ethical treatment of the study participants. This information sheet explained the study procedures, potential risks, and participants' rights.

#### 2.2.6 Statistical Analysis

The ice cream formulations were produced in two independent batches as experimental replicates, and all analytical measurements were performed in triplicate. Results are expressed as mean ± standard deviation. For microbiological properties, pH, color, and textural parameters, analysis of variance (ANOVA) was applied by including treatment, storage time, and the treatment × storage time interaction in the statistical model. For total phenolic content, sensory properties, and organic acid composition, comparisons were made only among treatments. When significant differences were observed, Fisher's LSD test was used for mean comparisons. Statistical significance was accepted at *p* < 0.01 and *p* < 0.05. The *p*‐values were reported in the corresponding tables. PCA was performed using the mean values obtained from textural and sensory analyses, and Pearson correlation analysis was performed using the mean values of all analyses using OriginPro 2024 software (OriginLab Corporation, Northampton, MA, USA).

## 3 Results and Discussion

### 3.1 Bacterial Viability and Post Acidification

Table 1 shows the changes in the counts of *B. animalis* subsp. *lactis* and *L. gasseri* in probiotic ice cream samples during storage. When live microorganisms are evaluated in terms of probiotic products, it is stated that probiotics should remain alive above the critical threshold (10^6^ cfu/g) in fermented foods throughout their shelf life to benefit consumer health (Tripathi and Giri 2014). When Table 1 is examined, it is determined that the number of bacteria required for the therapeutic effect is reached throughout the storage and in terms of varieties. Although the change in *B. animalis subsp. Lactis and L. gasseri* were not very obvious; the symbiotic system in which bacteria generally developed together showed a synergistic effect on the number and viability of both bacteria (*p* < 0.01). In addition, this effect is also seen during storage, and the bacterial count is maintained more steadily during 60‐day storage. In this study, probiotic *B. animalis* subsp. *lactis* and *L. gasseri* strains were added to the milk/ice cream mix, and the tolerance of bacteria to cold stress was determined by calculating bacterial viability before freezing (in the mix), after freezing, and during storage (1st and 2nd months). In addition, a system containing prebiotic/phenolic components (cocoa) was created, and the cell's cold adaptation process was monitored, along with the development/vitality activating effect of phenolic‐based cryoprotective compounds. Viability parameters in probiotic ice cream samples are given (*p* < 0.05) in Table 2. In sample B containing *B. animalis* subsp. *lactis*, the viability between VI_30–60_ and VI_1–60_ was higher than in other periods, but it had similar viability rates after freezing and during 30‐day storage. In sample C containing *L. gasseri* , bacterial viability was preserved during the 60‐day storage period. In matrix D, which contains both bacteria symbiotically, the number of *B. animalis* subsp. *lactis*, and *L. gasseri* remained stable after freezing during the 60‐day storage period. Gonzalez et al. (2008) stated that complex neutral oligosaccharides are prebiotic factors that stimulate bifidobacterial growth. It is understood that *Bifidobacterium* spp., like other living organisms, can activate complex molecular mechanisms to cope with heat stress and protect the cell from damage caused by the accumulation of unfolded and/or misfolded proteins. Many of these protective proteins include GroEL (Hsp60), DnaK (Hsp70), and ClpB (Hsp100), which play key roles in post‐translational events to prevent protein denaturation, aggregation, and misfolding (Wickner et al. 1999; Ventura et al. 2007). *L. gasseri* is effective in the fermentation of prebiotic fructans with fucosyltransferase enzymes that catalyze the conversion of sucrose to sugar and is also tolerant to low pH (Selle and Klaenhammer 2013; Ni et al. 2018). The effects of freezing, such as cell membrane lipid oxidation and cell damage, can reduce probiotic viability. Potential prebiotic components and co‐cultures can be used to prevent this cell loss, reduce the effects of cold stress, and maintain the therapeutic effect (Jouki et al. 2021). Massot‐Cladera et al. (2015) stated that other cocoa compounds, such as polyphenols or methylxanthines, which change the pH in different systems and affect SCFA formation, might alter the prebiotic effect of cocoa fibers. Salem et al. (2005) found that the initial freezing and subsequent hardening of the ice cream mixture caused a decrease of less than one log cycle in the number of live probiotics reported. During 12 weeks of frozen storage, a 1.68 and 1.23log_10_ cfu g^−1^ reduction was detected in probiotic bacteria, respectively. Table 3 presents the results of the variance analysis of the pH values of the samples (*p* < 0.05). The effect of lactic acid bacteria on acidity development during fermentation is known. It was determined that there were lower pH values and fermentation effects in the samples containing *L. gasseri*. However, no significant difference was detected in the pH values during storage.

**TABLE 1:** Results of variance analysis of bacterial counts of probiotic ice cream samples (log_10_ cfu g^−1^)*.

Samples	Storage (Days)	*B. animalis* subsp. *lactis*	*L. gasseri*
B	1	8.51 ± 0.034^a^	—
	30	8.02 ± 0.015^b^	—
	60	8.57 ± 0.072^a^	—
C	1	—	8.51 ± 0.168^a^
	30	—	8.00 ± 0.007^b^
	60	—	8.56 ± 0.005^a^
D	1	8.94 ± 0.153^a^	8.45 ± 0.989^a^
	30	8.48 ± 0.046^b^	8.48 ± 0.005^a^
	60	8.42 ± 0.114^b^	8.41 ± 0.143^a^

*Note:* B: ice cream containing *Bifidobacterium animalis* subsp. *lactis*; C: ice cream containing *Lactobacillus gasseri* ; D: ice cream containing *Bifidobacterium animalis* subsp. *lactis* + *Lactobacillus gasseri* .

* Averages with different letters are different from each other.

**TABLE 2:** Bacterial viability in probiotic ice cream samples.

Samples	Bacteria count					Viability parameters			
	Before freezing (BF) (log_10_ cfu g^−1^)	After freezing (AF) (log_10_ cfu g^−1^)	1^st^ day (log_10_ cfu g^−1^)	30^th^ day (log_10_ cfu g^−1^)	60^th^ day (log_10_ cfu g^−1^)	VI_AF‐BF_	VI_1_st_−30_th	VI_30_^th^_−60_th	VI_1_st_−60_th
B	8.79	8.71	8.51	8.02	8.57	0.99^ab^	0.94^ab^	1.06^a^	1.01^a^
C	10.11	9.88	8.51	8.00	8.56	0.98^ab^	0.94^b^	1.07^a^	1.00^a^
D ( *B. animalis* subsp. *lactis*)	9.34	9.16	8.94	8.48	8.42	0.98^a^	0.95^ab^	0.99^a^	0.94^ab^
D ( *L. gasseri* )	9.27	8.81	8.45	8.48	8.41	0.95^ab^	1.00^a^	0.99^a^	0.99^a^
*p*	—	—	—	—	—	**	**	**	**

*Note:* A: control ice cream; B: ice cream containing *Bifidobacterium animalis* subsp. *lactis*; C: ice cream containing *Lactobacillus gasseri* ; D: ice cream containing *Bifidobacterium animalis* subsp. *lactis* + *Lactobacillus gasseri* . Viability parameters according to process and storage time; VI = Final cell population (log_10_ cfu g^−1^)/ Initial cell population (log_10_ cfu g^−1^). (*) Significant at *p* < 0.05 level. (**) Significant at *p* < 0.01 level. Averages with different letters are different from each other.

**TABLE 3:** Results of variance analysis of pH values of probiotic ice cream samples*.

Samples	Storage (Days)	pH
A	1	6.29 ± 0.127^aA^
	30	6.25 ± 0.042^aA^
	60	6.29 ± 0.007^aA^
B	1	5.75 ± 0.007^bB^
	30	5.91 ± 0.028^aA^
	60	6.03 ± 0.028^bA^
C	1	5.64 ± 0.028^bA^
	30	5.78 ± 0.240^aA^
	60	5.94 ± 0.007^cA^
D	1	5.86 ± 0.177^bA^
	30	5.84 ± 0.163^aA^
	60	6.01 ± 0.007^bcA^

*Note:* A: control ice cream; B: ice cream containing *Bifidobacterium animalis* subsp. *lactis*; C: ice cream containing *Lactobacillus gasseri* ; D: ice cream containing *Bifidobacterium animalis* subsp. *lactis* + *Lactobacillus gasseri* .

* Averages with different letters are different from each other. Lower case letters indicate differences between probiotic ice cream samples, and upper case letters indicate differences between storage times.

**FIGURE 2:**
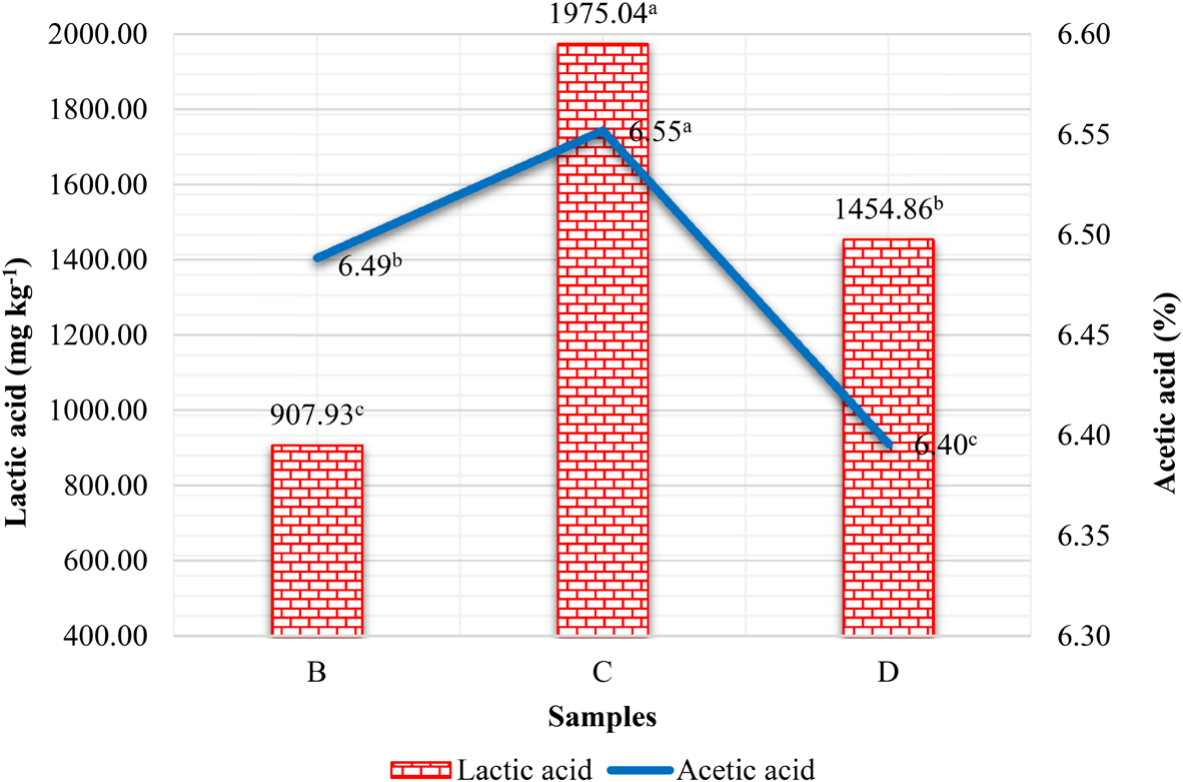
Lactic and acetic acid values of probiotic ice cream samples^†^. B: Ice cream containing *Bifidobacterium animalis* subsp. *lactis*; C: Ice cream containing *Lactobacillus gasseri* ; D: Ice cream containing *Bifidobacterium animalis* subsp. *lactis* + *Lactobacillus gasseri* . ^†^Averages with different letters are different from each other.

During fermentation, lactic acid bacteria primarily produce lactic acid and other organic substances. During this kinetic change, acids such as formic and acetic acid, as well as various fermentation by‐products (diacetyl, acetone, acetaldehyde, and antibacterial substances) that cause effective changes in the texture and aroma of dairy products, are also formed (Salem et al. 2005). Lactic and acetic acid values of probiotic ice cream samples are given in Figure 2. Lactic acid and acetic acid rates were determined to be higher in the C sample containing *L. gasseri* (*p* < 0.01). The ability of each bacterial strain to use potential substrates in the environment and their activities and metabolisms were practical here. While probiotics provide health benefits to the host when applied in adequate amounts, prebiotics positively affect host health by causing specific changes in the composition and/or activity of the gut microbiota. This matrix, which consists of substrates that are selectively used by living host microorganisms and provide health benefits to the host, creates a synbiotic effect. These synbiotic systems, called complementary, enable the formation of many metabolic substances by probiotic fermentation. These are compounds called postbiotics. Postbiotics are mixtures of non‐living microorganisms and/or their components and bioactive metabolites that provide health benefits to the host. Organic acid compounds such as lactic and acetic acid are also in this group (Mohanty et al. 2018; de Vrese and Schrezenmeir 2008; Hill et al. 2014; Walait et al. 2022). Researchers have reported that some phenolic compounds have prebiotic and SCFA‐forming effects (Karaman and Ozcan 2021; de Vos et al. 2022). Massot‐Cladera et al. (2015) and Sorrenti et al. (2020) also stated that cocoa has a prebiotic effect.

Similarly, it is stated that cocoa polyphenols modulate microbial diversity by promoting development. The ratio and type of polyphenols' antibacterial effect and prebiotic mechanisms' effect in different systems should be examined in detail (Gibson et al. 2017; Sorrenti et al. 2020; Sonnenburg and Sonnenburg 2014). It is stated that cocoa flavanols contribute prebiotically to maintaining the intestinal microbiota immunomodulation process (Kumar Singh et al. 2019).

Puertollano et al. (2014) and Tan et al. (2014) state that cocoa‐soluble and insoluble fibers have significant effects on host health by providing high levels of SCFA (acetic, propionic, and butyric acids) formation. A large portion of cocoa fibers consists of soluble and fermentable pectic substances. Cocoa is also a rich source of insoluble and less fermentable cellulose and hemicellulose (Tan et al. 2014). In general, cocoa fiber and polyphenol‐free composition support the development of beneficial bacteria (Lecumberri et al. 2007; Ni et al. 2018). In this study, it can be thought that cocoa components, although small, may affect bacterial development and fermentation.

### 3.2 Physicochemical Properties

Table 4 shows the changes in the color values of probiotic ice cream samples during storage. Changes in L*, a*, b*, and BI* parameters were detected. No significant difference in color values was observed during storage. Brightness was lower in the sample (B) containing *B. animalis* subsp. *lactis* (*p* < 0.05). The yellowness value was lower in sample D (*p* < 0.05). a* and BI* values did not change in general (*p* > 0.05). The transformation of plant substrates during fermentation forms compounds such as bioactive peptides, SCFA, and polysaccharides while also causing color degradation (Karaman and Ozcan 2021; Massot‐Cladera et al. 2014).

**TABLE 4:** Results of variance analysis of color properties of probiotic ice cream samples*.

Samples	Storage (Days)	L*	a*	b*	BI*
A	1	51.32 ± 0.0332^abAB^	8.58 ± 0.205^Aa^	17.62 ± 0.700^aB^	11.81 ± 0.202^aA^
	30	52.48 ± 0.007^aA^	9.14 ± 0.064^aA^	19.07 ± 0.028^aA^	12.29 ± 0.084^abA^
	60	48.85 ± 1.393^aB^	8.78 ± 0.460^aA^	16.51 ± 0.007^abB^	12.67 ± 0.986^aA^
B	1	48.71 ± 1.358^bA^	8.91 ± 0.665^aA^	17.73 ± 1.662^aA^	12.88 ± 0.587^aA^
	30	48.60 ± 0.594^bA^	9.61 ± 0.389^aA^	18.20 ± 0.686^aA^	13.88 ± 0.379^aA^
	60	46.70 ± 1.379^aA^	8.32 ± 0.502^aA^	15.31 ± 0.290^cA^	12.56 ± 1.089^aA^
C	1	54.66 ± 0.806^aA^	8.39 ± 0.156^aA^	18.05 ± 0.686^aA^	10.88 ± 1.151^aA^
	30	54.06 ± 0.403^aA^	8.38 ± 0.445^aA^	16.67 ± 0.940^aA^	10.99 ± 0.648^bA^
	60	50.16 ± 1.336^aB^	8.50 ± 0.481^aA^	16.70 ± 0.431^aA^	11.98 ± 0.963^aA^
D	1	55.21 ± 2.722^aA^	8.43 ± 0.509^aA^	17.91 ± 0.170^aA^	10.85 ± 1.151^aA^
	30	53.28 ± 0.057^aA^	9.14 ± 0.120^aA^	18.41 ± 0.375^aA^	12.11 ± 0.138^bA^
	60	51.11 ± 0.559^aA^	8.63 ± 0.042^aA^	15.86 ± 0.014^bcB^	11.92 ± 0.070^aA^

*Note:* A: control ice cream; B: ice cream containing *Bifidobacterium animalis* subsp. *lactis*; C: ice cream containing *Lactobacillus gasseri* ; D: ice cream containing *Bifidobacterium animalis* subsp. *lactis* + *Lactobacillus gasseri* .

* Averages with different letters are different from each other. Lower case letters indicate differences between probiotic ice cream samples, and upper case letters indicate differences between storage times.

The results of the variance analysis of phenolic compounds and antioxidant properties of probiotic ice cream samples are given in Table 5. It has been stated that lactic acid bacteria's potential to increase plant materials' antioxidant activity is due to decreased pH and the enzymolysis effect during fermentation (Terefe and Augustin 2019). TPC (mg GAE 100 mL^−1^) was determined to be the highest in sample C (*p* < 0.01). Studies show that fermentation using mixed strains rather than a single strain creates better biodegradation (Zhao et al. 2021). In general, in terms of antioxidant properties, no significant changes were detected in CUPRAC (mg TE 100 mL^−1^, *p* < 0.01) and DPPH (mg TE 100 mL^−1^, *p* > 0.05) values, even depending on the strain. Liu et al. (2017) reported that antioxidant activity increased due to allowing antioxidant phenolics and increased bioavailability of free hydroxyl groups during fermentation. However, Dulf et al. (2016) reported a slight decrease in free phenolic content after fermentation, probably due to oxidative enzymes polymerizing the released phenolics in bub. Ricci et al. (2019) stated that the metabolism of phenolics depends on the strain characteristics and species. It was also noted that fermentation advanced the radical scavenging activity of black tea and increased the metal chelating activity of DPPH during the 24‐h fermentation period. In general, it is stated that fermentation plays an essential role in improving antioxidant activity (Terefe and Augustin 2019). However, no primary biochemical reaction occurred in this study since the milk matrix was added to the ice cream mix after fermentation. Therefore, the TPC and antioxidant capacity in samples containing equal amounts of cocoa did not create significant changes.

**TABLE 5:** Results of variance analysis of phenolic compounds and antioxidant properties of probiotic ice cream samples.

Samples	TPC (mg GAE 100 ml^−1^)	CUPRAC (mg TE 100 ml^−1^)	DPPH (mg TE 100 mL^−1^)
A	36.68 ± 1.800^b^	181.72 ± 11.677^c^	9.42 ± 0.763^a^
B	35.04 ± 0.508^b^	170.06 ± 7.469^c^	8.98 ± 0.635^a^
C	44.08 ± 0.809^a^	231.17 ± 8.457^b^	8.90 ± 0.420^a^
D	36.36 ± 1.606^b^	283.11 ± 6.255^a^	8.93 ± 0.956^a^
*p*	**	**	ns

*Note:* A: control ice cream; B: ice cream containing *Bifidobacterium animalis* subsp. *lactis*; C: ice cream containing *Lactobacillus gasseri* ; D: ice cream containing *Bifidobacterium animalis* subsp. *lactis* + *Lactobacillus gasseri* . (**) Significant at *p* < 0.01 level. (ns) Not significant. Averages with different letters are different from each other.

### 3.3 Textural Properties

The structural network of foods greatly influences sensory acceptance. Table 6 shows the results of the variance analysis of the textural properties of probiotic ice cream samples. Although firmness values did not create significant differences in terms of samples, firmness (*p* < 0.01) and, accordingly, work of penetration (*p* < 0.01) increased during storage. No significant changes were detected in adhesiveness values depending on samples (*p* < 0.05) and storage period (*p* > 0.05).

**TABLE 6:** Results of variance analysis of textural properties of probiotic ice cream samples*.

Samples	Storage (Days)	Firmness (g)	Penetration (g.s)	Adhesiveness (g.s)
A	1	449.47 ± 25.452^aC^	1663.18 ± 150.368^aB^	35.95 ± 8.378^aA^
	30	1966.45 ± 167.402^aB^	3596.83 ± 224.388^abA^	37.32 ± 7.180^bA^
	60	2889.81 ± 153.305^aA^	4235.01 ± 503.307^bA^	45.94 ± 5.714^aA^
B	1	488.81 ± 91.069^aC^	1623.10 ± 212.106^aB^	37.15 ± 2.450^aA^
	30	1305.89 ± 27.955^bB^	3328.10 ± 276.755^bA^	40.76 ± 4.140^bA^
	60	2336.81 ± 304.191^aA^	4002.66 ± 338.388^bA^	42.65 ± 11.578^aA^
C	1	386.23 ± 1.875^aC^	417.00 ± 30.605^bB^	49.26 ± 6.670^aA^
	30	1353.31 ± 144.328^bB^	2976.08 ± 153.438^bB^	66.16 ± 18.230^aA^
	60	3580.52 ± 222.819^aA^	7681.80 ± 151.653^aA^	44.82 ± 12.762^aA^
D	1	215.81 ± 2.510^aB^	733.07 ± 70.538^bB^	50.31 ± 10.130^aA^
	30	2056.97 ± 153.148^aAB^	4571.14 ± 458.390^aA^	51.06 ± 10.865^aA^
	60	3520.02 ± 128.891^aA^	6546.79 ± 154.432^aA^	57.66 ± 15.005^aA^

*Note:* A: control ice cream; B: ice cream containing *Bifidobacterium animalis* subsp. *lactis*; C: ice cream containing *Lactobacillus gasseri* ; D: ice cream containing *Bifidobacterium animalis* subsp. *lactis* + *Lactobacillus gasseri* .

* Averages with different letters are different from each other. Lower case letters indicate differences between probiotic ice cream samples, and upper case letters indicate differences between storage times.

**FIGURE 3:**
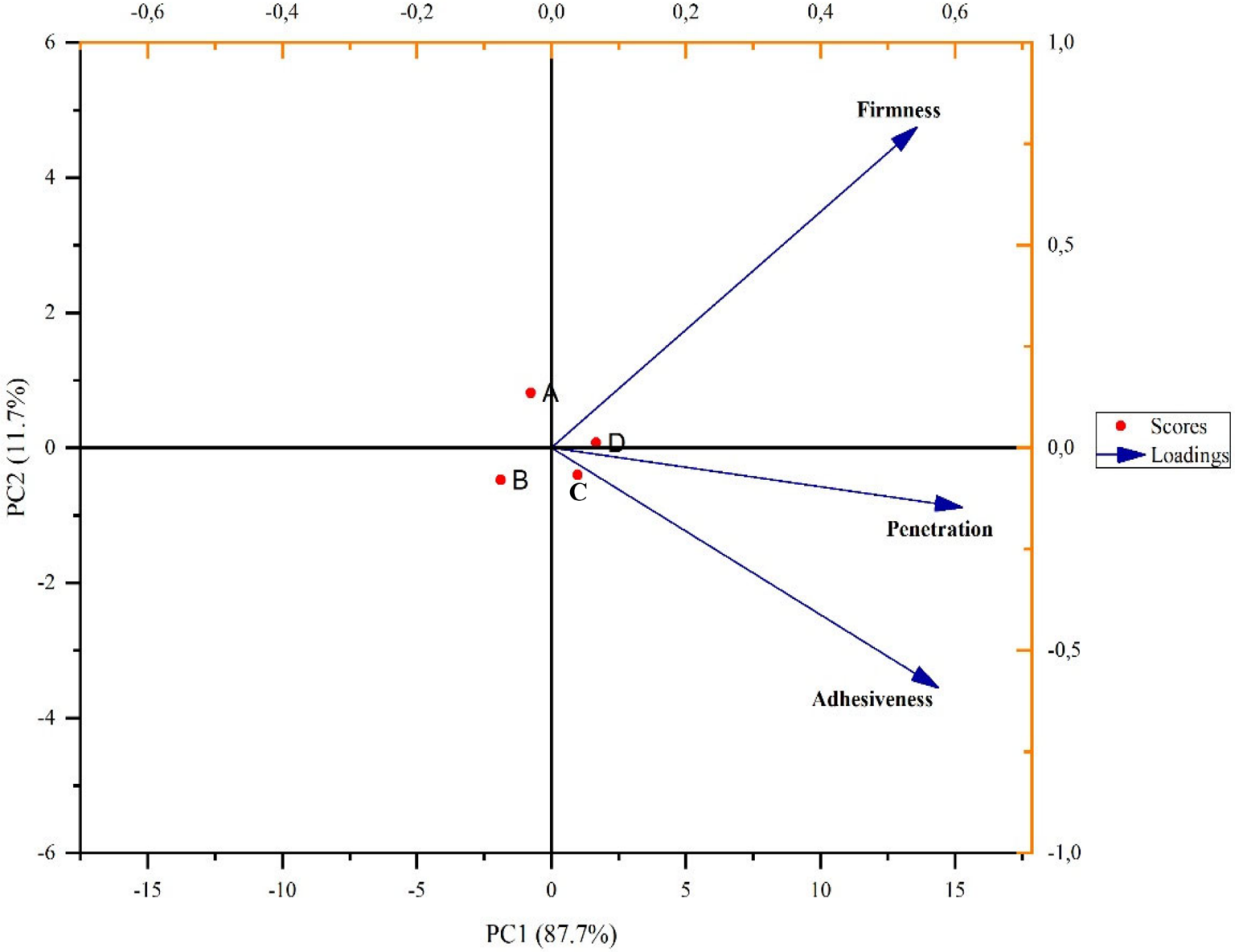
Biplot graph of textural properties of probiotic ice cream samples. A: Control ice cream; B: Ice cream containing *Bifidobacterium animalis* subsp. *lactis*; C: Ice cream containing *Lactobacillus gasseri* ; D: Ice cream containing *Bifidobacterium animalis* subsp. *lactis* + *Lactobacillus gasseri* .

The PCA graph of the textural properties of probiotic ice cream samples is given in Figure 3. PC1 explains 87.7% of the total variance, and PC2 explains 11.7%. Control (A) and B (containing *B. animalis* subsp. *lactis*) samples are located in the negative region of PC1 in the biplot graph, indicating that the samples are different from each other. C (containing *L. gasseri* ) and D (containing *B. animalis* subsp. *lactis* and *L. gasseri* ) samples are closer to the center of the graph and in the positive region of PC1, indicating a positive similarity in terms of the textural properties of the samples. It was determined that the firmness loadings had a high positive value and that C and D samples in the same region came to the fore with this textural property. Penetration is positioned positively according to PC1, indicating that C and D samples may have higher penetration values. Adhesiveness is placed in a negative and PC2 direction, indicating that this property will create a significant difference in B and C samples.

The examined textural parameters were generally determined to be higher in the multi‐strain system containing two bacteria. El‐Nagar et al. (2002) stated that ice cream mix composition, gelation properties, and increased water binding capacity of proteins with high solute concentrations create changes in freezing points, increasing the ice cream's viscosity and changing the mixture's rheology. Mohammadi et al. (2011) also stated that supplementing ice cream with probiotic bacteria has minimal effect on the taste, texture, or other sensory properties of ice cream.

### 3.4 Hedonic and Descriptive Analysis of Samples for Consumer Testing

Acceptance or liking tests are applied to assess consumer preference for a product. The results of consumer liking and sensory values of probiotic ice cream samples are given in Table 7. At the beginning of storage, sample D containing both bacteria received lower scores for all hedonic sensory parameters in general, with more pronounced changes in terms of appearance and structural properties. At the end of storage, it was determined that the liking scores of ice creams containing probiotic bacteria increased. Samples B and C are noted as smoother. Sample B was generally more appreciated at the beginning and end of storage, particularly in terms of many sensory properties and overall acceptability. The best melting quality was in samples A and B, while the cold feeling was preferred in sample A. In terms of purchasing, the traditional one and the B example were preferred more. Although sample D was less liked in terms of taste characteristics, it was in the “liked it‐liked it very much” group on the hedonic scale with values of 4.13–4.43 (Table 7). In general, including prebiotic ingredients in ice cream significantly affects flavor and texture, while fermentation of probiotics primarily affects sensory and aromatic properties (Cruz et al. 2010). Eroglu and Ozcan (2024) also documented no difference in overall liking for diet yogurts sweetened with non‐nutritive sweetener‐stevia, which was used as a potential prebiotic, compared to the control samples. It has been stated that the metabolism of probiotic cultures, especially the molar ratio of acetic acid formed by the *Bifidobacterium* fermentation pathway to lactic acid, may cause a taste difference. Therefore, it is essential to investigate multiple formulations and co‐cultures in the production of probiotic ice creams with high sensory acceptance (Mohammadi et al. 2011; Tamime et al. 2005; Aryana and Summers 2006).

**TABLE 7:** Consumer appreciation values of probiotic ice cream samples at the beginning and of freezing storage (−18°C).*

	Samples	Sensory analysis parameters											
		Appearance	Structure	Smoothness	Odor	Taste	Color	Aroma intensity	Melting quality	Cold feeling	Aftertaste	General acceptability	Purchase intention
Beginning of storage	A	4.83 ± 0.206^a^	4.73 ± 0.222^b^	4.68 ± 0.457^b^	4.65 ± 0.238^a^	4.88 ± 0.250^a^	4.80 ± 0.245^b^	4.68 ± 0.472^b^	4.78 ± 0.263^a^	4.88 ± 0.250^a^	4.60 ± 0.271^b^	4.68 ± 0.236^b^	4.75 ± 0.208^a^
	B	4.88 ± 0.122^a^	4.93 ± 0.195^a^	5.00 ± 0.000^a^	4.50 ± 0.548^b^	4.63 ± 0.447^b^	5.00 ± 0.000^a^	5.00 ± 0.000^a^	4.73 ± 0.438^a^	4.68 ± 0.422^b^	4.95 ± 0.089^a^	4.75 ± 0.447^a^	4.63 ± 0.447^ab^
	C	4.93 ± 0.173^a^	4.64 ± 0.503^b^	4.74 ± 0.231^b^	4.73 ± 0.577^a^	4.58 ± 0.451^b^	4.82 ± 0.289^b^	4.63 ± 0.404^b^	4.60 ± 0.513^b^	4.66 ± 0.321^b^	4.26 ± 0.764^c^	4.53 ± 0.529^c^	4.49 ± 0.577^c^
	D	4.55 ± 0.416^b^	4.00 ± 0.756^c^	4.38 ± 0.427^c^	4.33 ± 0.508^c^	4.13 ± 0.570^c^	4.38 ± 0.500^c^	4.43 ± 0.378^c^	4.38 ± 0.447^c^	4.30 ± 0.422^c^	4.25 ± 0.447^c^	4.35 ± 0.438^d^	4.25 ± 0.447^d^
End of storage	A	4.53 ± 0.492^c^	4.88 ± 0.200^b^	4.65 ± 0.383^b^	4.90 ± 0.163^a^	4.93 ± 0.098^a^	4.70 ± 0.400^b^	4.88 ± 0.186^a^	4.90 ± 0.167^a^	5.00 ± 0.041^a^	4.70 ± 0.392^a^	4.85 ± 0.176^b^	4.95 ± 0.103^b^
	B	5.00 ± 0.000^a^	4.95 ± 0.103^a^	4.75 ± 0.197^a^	4.90 ± 0.163^a^	4.93 ± 0.082^a^	4.78 ± 0.321^b^	4.83 ± 0.204^a^	4.70 ± 0.388^b^	4.75 ± 0.408^b^	4.55 ± 0.438^b^	4.93 ± 0.084^a^	4.98 ± 0.041^a^
	C	4.50 ± 0.516^c^	4.70 ± 0.389^c^	4.70 ± 0.190^a^	4.95 ± 0.082^a^	4.70 ± 0.331^b^	4.70 ± 0.400^b^	4.78 ± 0.235^b^	4.50 ± 0.505^c^	4.63 ± 0.418^c^	4.58 ± 0.390^b^	4.63 ± 0.378^c^	4.53 ± 0.378^b^
	D	4.70 ± 0.400^b^	4.75 ± 0.313^c^	4.35 ± 0.463^c^	4.95 ± 0.082^a^	4.43 ± 0.449^c^	4.83 ± 0.204^a^	4.38 ± 0.501^c^	4.50 ± 0.420^c^	4.63 ± 0.418^c^	4.55 ± 0.415^b^	4.60 ± 0.333^c^	4.55 ± 0.387^b^

*Note:* A: control ice cream; B: ice cream containing *Bifidobacterium animalis* subsp. *lactis*; C: ice cream containing *Lactobacillus gasseri* ; D: ice cream containing *Bifidobacterium animalis* subsp. *lactis* + *Lactobacillus gasseri* .

* Averages with different letters are different from each other.

**FIGURE 4:**
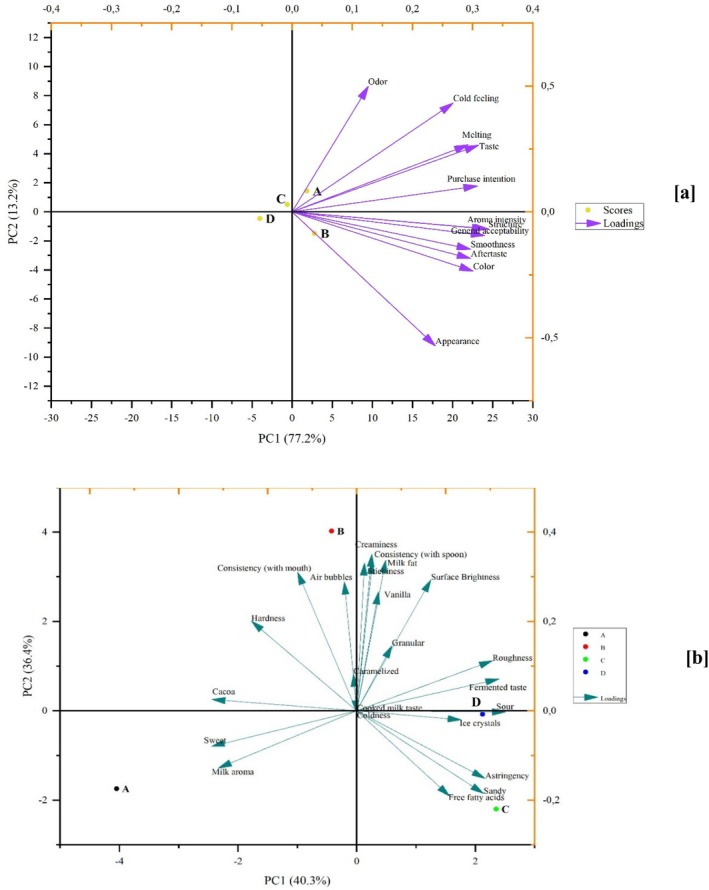
Biplot graph of hedonic [a] and QDA [b] results of probiotic ice cream samples. A: Control ice cream; B: Ice cream containing *Bifidobacterium animalis* subsp. *lactis*; C: Ice cream containing *Lactobacillus gasseri* ; D: Ice cream containing *Bifidobacterium animalis* subsp. *lactis* + *Lactobacillus gasseri* .

The PCA graph of the sensory properties of probiotic ice cream samples evaluated on the hedonic scale is given in Figure 4a. PC1 explains 77.2% of the total variance, while PC2 explains 13.2%. The A and B samples are similar in terms of the definitions given on the hedonic scale, while the C and D samples have differences regarding the same sensory definitions. In addition, the D sample contains *B. animalis* subsp. *lactic* and *L. gasseri* are in the negative region, showing lower liking scores. The odor feature has a distinguishing effect among the samples. That cold feeling, melting, taste, and purchase intention have higher liking scores in the A and B samples, which are in the positive region. These samples positively correlated with the aroma intensity, general acceptability, smoothness, aftertaste, and color features. The fact that the appearance parameter is high and in the negative region shows that there may be a negative distinguishing feature between the samples.

The PCA graph of the QDA results of probiotic ice cream samples is given in Figure 4b. PC1 explains 40.3% of the total variance, while PC2 explains 36.4% of the total variance. The A sample stands out in terms of its cocoa, sweetness, and milk flavor characteristics, and it is in the negative region of PC1 and PC2 components. This shows that the A sample has a sweeter and milky profile. Sample B, which contains *B. animalis* subsp. *animalis*, is more associated with consistency (with the mouth), air bubbles, and hardness. This indicates that the B sample has a more rigid structure and air bubbles. The C sample, which contains *L. gasseri* , was determined to be associated with negative sensory characteristics such as astringency, sandy, and free fatty acids. The taste profile of this sample was perceived as lower due to the mentioned characteristics. The D sample is defined by a more sour taste and a coldness perception. The use of sugar replacements in fermented products not only masks the sour taste but also affects the texture and flavor characteristics of the final product. Moreover, consumers expect the sensory quality to be similar to the full‐sugar forms, correlated with other sensory profiles (Fávaro‐Trindade et al. 2007; Torrico et al. 2020; Eroglu and Ozcan 2024). Li et al. (2015) observed bitterness and astringency in stevia‐sweetened chocolate milk, and Keefer et al. (2020) detected bitterness and metallic taste in stevia‐sweetened protein bars. However, these sensory descriptors were not detected in probiotic ice creams in our study. Probiotic fermentation and acidity development affected the appearance or perception of off‐flavors and chemical aftertastes.

### 3.5 The Interaction Between the Microbiological, Physicochemical and Sensorial Analysis Results

**FIGURE 5:**
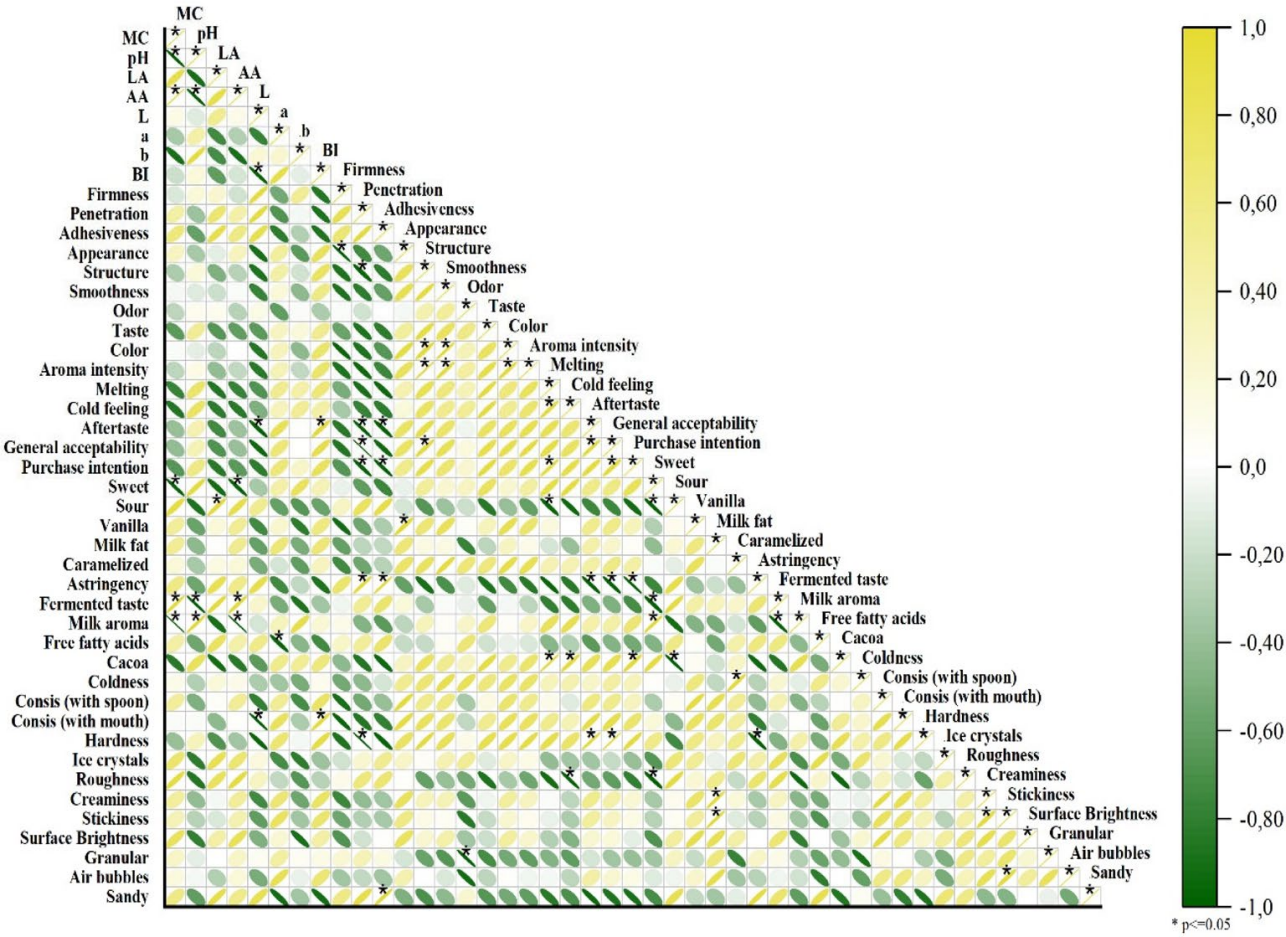
Pearson correlation plot of microbiological, physicochemical, and sensorial results of probiotic ice cream samples (Correlation coefficients are indicated by the color green: Negative correlation; yellow: Positive correlation. MC: Microorganisms count; LA: Lactic acid; AA: Acetic acid). (*) Significant at *p* < 0.05 level.

The Pearson correlation graph, which was performed to explain the interaction between the results of microbiological, physicochemical, and sensory (hedonic and QDA) properties of probiotic ice cream samples, is given in Figure 5. Yellow color indicates a positive correlation between the properties, and green color indicates a negative correlation. The number of microorganisms in the samples positively correlated with lactic acid, penetration, adhesiveness, sourness, and fermented milk taste. Milk fat and creaminess were also positively correlated, and as milk fat increases, the creaminess sensation may also increase. It was determined that the perception of sweetness and vanilla in ice creams positively affected general acceptability. In contrast, the presence of low levels of ice crystals perceived by some panelists negatively affected general acceptability. This correlation matrix reveals the relationship between the parameters that should be controlled in creating probiotic ice cream formulations, determining consumer preferences, and developing quality strategies.

## 4 Conclusion

Recently, fermented dairy products and desserts have attracted attention as alternative matrices for developing probiotic bacteria. The ice cream matrix is a suitable substrate for probiotic cultures because it contains milk proteins, fat, lactose, and other compounds. Moreover, being a frozen product and its high pH values (5.5 to 6.5) contribute to the survival of these beneficial cultures. In this study, it was determined that the growth‐activating effect was higher in sample D, in which the two bacteria were present together, and higher counts were reached compared to the systems in which the bacteria were present individually. The produced ice cream contained biotherapeutic levels of bacteria (8.00 ≤ log10 cfu g^−1^). Postbiotic organic acids were higher in sample C ( *L. gasseri* ). In addition, the produced ice cream showed antioxidant‐rich, textural, and sensory properties. This study also determined that creating a synbiotic correlation matrix can identify the relationships among the characteristics that should be controlled in determining consumer preferences and developing quality strategies in probiotic ice cream formulations. It is believed that this study may provide new insights into potential applications related to the resistance mechanisms of probiotic bacteria in cold‐frozen systems and contribute to the development of innovative frozen functional foods. Furthermore, this study presents the evaluation of *Bifidobacterium animalis* subsp. *lactis* and *Lactobacillus gasseri* in ice cream containing phenolic stevia.

## Author Contributions

**Tulay Ozcan:** funding acquisition, writing – original draft, writing – review and editing, investigation, conceptualization, methodology, project administration, supervision, validation. **Gokce Keser:** formal analysis, conceptualization, writing – original draft, software, data curation. **Ali Riza Kaya:** formal analysis, conceptualization, investigation, data curation. **Yunus Emre Cetin:** formal analysis, data curation, investigation. **Fatime Elciboga:** data curation, formal analysis, investigation.

## Funding

The present study was funded by TUBITAK (The Scientific and Technological Research Council of Turkiye) (Project: 2209‐A, 2024).

## Ethics Statement

In this study, the sensory analysis performed involved adult volunteers and did not include any medical, invasive, or clinical procedures. Therefore, in accordance with national legislation and institutional guidelines, formal ethical approval was not required.

## Consent

Prior to participation, all panelists were informed about the study objectives and procedures, and their informed consent was obtained. Participation was voluntary, and participants had the right to withdraw at any time without any consequence. No personal or sensitive data were recorded, and all responses were kept anonymous and confidential.

## Conflicts of Interest

The authors declare no conflicts of interest.

## Data Availability

The data that support the findings of this study are available on request from the corresponding author. The data are not publicly available due to privacy or ethical restrictions.

## References

[fsn372030-bib-0001] Al‐Hilphy, A. R. , H. I. Ali , S. A. Al‐IEssa , M. Gavahian , and A. Mousavi‐Khaneghah . 2022. “Assessing Compositional and Quality Parameters of Unconcentrated and Refractive Window Concentrated Milk Based on Color Components.” Dairy 3: 400–412.

[fsn372030-bib-0002] Aryana, K. J. , and M. Summers . 2006. “Probiotic Fat‐Free, no Sugar Added Ice Cream.” Milchwissenschaft 61: 84–187.

[fsn372030-bib-0003] Balthazar, C. F. , H. L. A. Silva , R. M. S. Celequini , et al. 2015. “Effect of Galacto Oligosaccharide Addition on the Physical, Optical, and Sensory Acceptance of Vanilla Ice Cream.” Journal of Dairy Science 98: 4266–4272. 25912870 10.3168/jds.2014-9018

[fsn372030-bib-0004] Bindels, L. B. , N. M. Delzenne , P. D. Cani , and J. Walter . 2015. “Towards a More Comprehensive Concept for Prebiotics.” Nature Reviews. Gastroenterology & Hepatology 12: 303–310. 25824997 10.1038/nrgastro.2015.47

[fsn372030-bib-0005] Birch, C. S. , and G. A. Bonwick . 2019. “Ensuring the Future of Functional Foods.” International Journal of Food Science and Technology 54: 1467–1485.

[fsn372030-bib-0006] Boyanova, P. , D. Gradinarska , V. Dobreva , P. Panayotov , M. Momchilova , and G. Zsivanovits . 2022. “Effect of *Spirulina platensis* on the Quality and Antioxidant Characteristics of Ice Cream.” BIO Web of Conferences 45: 1009.

[fsn372030-bib-0007] Crown, E. , D. Rovai , C. M. Racette , D. M. Barbano , and M. A. Drake . 2024. “Consumer Perception of Sweeteners in Yogurt.” Journal of Dairy Science 107: 10552–10570. 39245170 10.3168/jds.2024-24862

[fsn372030-bib-0008] Cruz, A. G. , A. E. C. Antunes , A. Sousa , J. A. F. Faria , and S. M. I. Saad . 2009. “Ice‐Cream as a Probiotic Food Carrier.” Food Research International 42: 1233–1239.

[fsn372030-bib-0009] Cruz, A. G. , R. S. Cadena , E. H. M. Walter , et al. 2010. “Sensory Analysis: Relevance for Prebiotic, Probiotic and Symbiotic Product Development.” Comprehensive Reviews in Food Science and Food Safety 9: 358–373. 33467838 10.1111/j.1541-4337.2010.00115.x

[fsn372030-bib-0010] de Souza, J. C. B. , M. d. R. Costa , C. M. V. B. de Rensis , and K. Sivieri . 2010. “Ice Cream: Composition, Processing and Addition of Probiotic.” Brazilian Journal of Food Technology 21: 155–165.

[fsn372030-bib-0011] de Vos, W. M. , H. Tilg , M. Van Hul , and P. D. Cani . 2022. “Gut Microbiome and Health: Mechanistic Insights.” Gut 71: 1020–1032. 35105664 10.1136/gutjnl-2021-326789 PMC8995832

[fsn372030-bib-0012] de Vrese, M. , and J. Schrezenmeir . 2008. “Probiotics, Prebiotics, and Synbiotics.” Advances in Biochemical Engineering/Biotechnology 111: 1–66. 18461293 10.1007/10_2008_097

[fsn372030-bib-0013] Dogan, S. , B. Kocaturk , S. Kara , M. E. Diken , and M. Dogan . 2021. “The Effects of Drying Methods and Gamma Irradiations on Total Phenolic and Flavonoid Amounts, Antioxidant Capacities and Phenolic Compound Contents of Olive Leaves.” Agrociencia 55: 10.

[fsn372030-bib-0014] Dulf, F. V. , D. C. Vodnar , and C. Socaciu . 2016. “Effects of Solid‐State Fermentation With Two Filamentous Fungi on the Total Phenolic Contents, Flavonoids, Antioxidant Activities and Lipid Fractions of Plum Fruit ( *Prunus domestica* L.) by‐Products.” Food Chemistry 209: 27–36. 27173530 10.1016/j.foodchem.2016.04.016

[fsn372030-bib-0015] El‐Nagar, G. , G. Clowes , C. M. Tudorică , V. Kuri , and C. S. Brennan . 2002. “Rheological Quality and Stability of Yog‐Ice Cream With Added Inulin.” International Journal of Dairy Technology 55: 89–93.

[fsn372030-bib-0016] Eroglu, E. , and T. Ozcan . 2024. “Pro‐Pre and Postbiotic Fermentation of the Dietetic Dairy Matrix With Prebiotic Sugar Replacers.” Probiotics and Antimicrobial Proteins 16: 726–736. 37093514 10.1007/s12602-023-10069-3

[fsn372030-bib-0017] Fávaro‐Trindade, C. S. , J. C. C. Balieiro , P. F. Dias , F. A. Sanino , and C. Boschini . 2007. “Effects of Culture, pH and Fat Concentration on Melting Rate and Sensory Characteristics of Probiotic Fermented Yellow Mombin ( *Spondias mombin* L.) Ice Creams.” Food Science and Technology International 13: 285–291.

[fsn372030-bib-0018] Ferraz, J. L. , A. G. Cruz , R. S. Cadena , et al. 2012. “Sensory Acceptance and Survival of Probiotic Bacteria in Ice Cream Produced With Different Overrun Levels.” Journal of Food Science 71: 25–28. 10.1111/j.1750-3841.2011.02508.x 22260128

[fsn372030-bib-0019] Figueroa‐González, I. , G. Quijano , G. Ramírez , and A. Cruz‐Guerrero . 2011. “Probiotics and Prebiotics—Perspectives and Challenges.” Journal of the Science of Food and Agriculture 91: 1341–1348. 21445871 10.1002/jsfa.4367

[fsn372030-bib-0020] Gibson, G. R. , R. Hutkins , M. E. Sanders , et al. 2017. “Expert Consensus Document: The International Scientific Association for Probiotics and Prebiotics (ISAPP) Consensus Statement on the Definition and Scope of Prebiotics.” Nature Reviews. Gastroenterology & Hepatology 14: 491–502. 28611480 10.1038/nrgastro.2017.75

[fsn372030-bib-0021] Goff, H. D. , and R. W. Hartel . 2013. Ice cream, 371. Springer.

[fsn372030-bib-0022] Gonzalez, R. , E. S. Klaassens , E. Malinen , W. M. de Vos , and E. E. Vaughan . 2008. “Differential Transcriptional Responses of *Bifidobacterium longum* to Human Milk, Formula Milk, and Galactooligosaccharide.” Applied and Environmental Microbiology 74: 4686–4694. 18539808 10.1128/AEM.00122-08 PMC2519361

[fsn372030-bib-0023] Gueimonde, M. , and B. Sanchez . 2012. “Enhancing Probiotic Stability in Industrial Processes.” Microbial Ecology in Health and Disease 23: 18562. 10.3402/mehd.v23i0.18562 PMC3747747 23990824

[fsn372030-bib-0024] Hill, C. , F. Guarner , G. Reid , et al. 2014. “The International Scientific Association for Probiotics and Prebiotics Consensus Statement on the Scope and Appropriate Use of the Term Probiotic.” Nature Reviews. Gastroenterology & Hepatology 11: 506–514. 24912386 10.1038/nrgastro.2014.66

[fsn372030-bib-0025] Holmes, Z. C. , J. D. Silverman , H. K. Dressman , et al. 2020. “Short‐Chain Fatty Acid Production by Gut Microbiota From Children With Obesity Differs According to Prebiotic Choice and Bacterial Community Composition.” MBio 11: 10–1128. 10.1128/mBio.00914-20 PMC7439474 32788375

[fsn372030-bib-0027] International Organization for Standardization . 2003. “ISO 4121:2003 Sensory Analysis – Guidelines for the Use of Quantitative Response Scales.” International Organization for Standardization, Geneva, Switzerland.

[fsn372030-bib-0026] International Organization for Standardization . 2016. “ISO 13299:2016. Sensory Analysis – Methodology – General Guidance for Establishing a Sensory Profile.” Geneva: International Organization for Standardization, Geneva, Switzerland.

[fsn372030-bib-0028] John, R. 2021. “Functional Foods: Components, Health Benefits, Challenges, and Major Projects.” DRC Sustainable Future: Journal of Environmental, Agricultural and Energy 2: 61–72.

[fsn372030-bib-0029] Jouki, M. , N. Khazaei , F. Rezaei , and R. Taghavian‐Saeid . 2021. “Production of Synbiotic Freeze‐Dried Yoghurt Powder Using Microencapsulation and Cryopreservation of *Lactobacillus plantarum* in Alginate Skim Milk Microcapsules.” International Dairy Journal 122: 105133.

[fsn372030-bib-0030] Karaman, S. , and T. Ozcan . 2021. “Determination of Gelation Properties and Bio‐Therapeutic Potential of Black Carrot Fibre‐Enriched Functional Yoghurt Produced Using Pectin and Gum Arabic as Prebiotic.” International Journal of Dairy Technology 74: 505–517.

[fsn372030-bib-0031] Keefer, H. R. M. , S. Nishku , P. D. Gerard , and M. A. Drake . 2020. “Role of Sweeteners on Temporality and Bar Hardening of Protein Bars.” Journal of Dairy Science 103: 6032–6053. 32448575 10.3168/jds.2019-17995

[fsn372030-bib-0032] Keser, G. , and T. Ozcan . 2025. “Cross‐Over Fermentation Dynamics and Proteomic Properties of Acid Gels With Indigenous *Lactobacillus* spp. Isolated From Cheeses.” Food Microbiology 128: 104700. 39952741 10.1016/j.fm.2024.104700

[fsn372030-bib-0033] Kumar Singh, A. , C. Cabral , R. Kumar , et al. 2019. “Beneficial Effects of Dietary Polyphenols on Gut Microbiota and Strategies to Improve Delivery Efficiency.” Nutrients 11: 2216. 31540270 10.3390/nu11092216 PMC6770155

[fsn372030-bib-0034] Lecumberri, E. , R. Mateos , M. Izquierdo‐Pulido , P. Rupérez , L. Goya , and L. Bravo . 2007. “Dietary Fibre Composition, Antioxidant Capacity and Physico‐Chemical Properties of a Fibre‐Rich Product From Cocoa ( *Theobroma cacao* L.).” Food Chemistry 104: 948–954.

[fsn372030-bib-0035] Lee, O. H. , B. Y. Lee , J. Lee , et al. 2009. “Assessment of Phenolics‐Enriched Extract and Fractions of Olive Leaves and Their Antioxidant Activities.” Bioresource Technology 100: 6107–6113. 19608415 10.1016/j.biortech.2009.06.059

[fsn372030-bib-0036] Li, X. E. , K. Lopetcharat , and M. A. Drake . 2015. “Parents' and Children's Acceptance of Skim Chocolate Milks Sweetened by Monk Fruit and Stevia Leaf Extracts.” Journal of Food Science 80: 1083–1092. 10.1111/1750-3841.12835 25847181

[fsn372030-bib-0037] Lis, A. , B. Staniewski , and J. Ziajka . 2021. “A Comparison of Butter Texture Measurements With the AP 4/2 Penetrometer and TA.XT Plus Texture Analyzer.” International Journal of Food Properties 24: 1744–1757.

[fsn372030-bib-0038] Liu, L. , R. Zhang , Y. Deng , et al. 2017. “Fermentation and Complex Enzyme Hydrolysis Enhance Total Phenolics and Antioxidant Activity of Aqueous Solution From Rice Bran Pretreated by Steaming With α‐Amylase.” Food Chemistry 221: 636–643. 27979252 10.1016/j.foodchem.2016.11.126

[fsn372030-bib-0039] Massot‐Cladera, M. , M. Abril‐Gil , S. Torres , À. Franch , M. Castell , and F. J. Pérez‐Cano . 2014. “Impact of Cocoa Polyphenol Extracts on the Immune System and Microbiota in Two Strains of Young Rats.” British Journal of Nutrition 112: 1944–1954. 25345541 10.1017/S0007114514003080

[fsn372030-bib-0040] Massot‐Cladera, M. , A. Costabile , C. E. Childs , et al. 2015. “Prebiotic Effects of Cocoa Fibre on Rats.” Journal of Functional Foods 19: 341–352.

[fsn372030-bib-0041] Mohammadi, R. , A. M. Mortazavian , R. Khosrokhavar , and A. G. Cruz . 2011. “Probiotic Ice Cream: Viability of Probiotic Bacteria and Sensory Properties.” Annales de Microbiologie 61: 411–424.

[fsn372030-bib-0042] Mohanty, D. , S. Misra , S. Mohapatra , and P. S. Sahu . 2018. “Prebiotics and Synbiotics: Recent Concepts in Nutrition.” Food Bioscience 26: 152–160.

[fsn372030-bib-0043] Mora, M. R. , Z. Wang , J. M. Goddard , and R. Dando . 2022. “Consumers Respond Positively to the Sensory, Health, and Sustainability Benefits of the Rare Sugar Allulose in Yogurt Formulations.” Food 11: 3718. 10.3390/foods11223718 PMC9689152 36429310

[fsn372030-bib-0044] Neri‐Numa, I. A. , H. S. Arruda , M. V. Geraldi , M. R. Marostica Júnior , and G. M. Pastore . 2020. “Natural Prebiotic Carbohydrates, Carotenoids and Flavonoids as Ingredients in Food Systems.” Current Opinion in Food Science 33: 98–107.

[fsn372030-bib-0045] Ni, D. , Y. Zhu , W. Xu , Y. Bai , T. Zhang , and W. Mu . 2018. “Biosynthesis of Inulin From Sucrose Using Inulosucrase From *Lactobacillus gasseri* DSM 20604.” International Journal of Biological Macromolecules 109: 1209–1218. 29169948 10.1016/j.ijbiomac.2017.11.120

[fsn372030-bib-0046] Ozcan, T. , T. Ozdemir , and H. R. Avci . 2021. “Survival of *Lactobacillus casei* and Functional Characteristics of Reduced Sugar Red Beetroot Yoghurt With Natural Sugar Substitutes.” International Journal of Dairy Technology 74: 148–160.

[fsn372030-bib-0047] Ozcan, T. , S. Sahin , A. Akpinar Bayizit , and L. Yilmaz Ersan . 2019. “Assessment of Antioxidant Capacity by Method Comparison and Amino Acid Characterisation in Buffalo Milk Kefir.” International Journal of Dairy Technology 72: 65–73.

[fsn372030-bib-0048] Ozcan‐Yilsay, T. , L. Yilmaz , and A. Akpinar‐Bayizit . 2006. “The Effect of Using a Whey Protein Fat Replacer on Textural and Sensory Characteristics of Low‐Fat Vanilla Ice Cream.” European Food Research and Technology 222, no. 1/2: 171–175.

[fsn372030-bib-0049] Pon, S. Y. , W. J. Lee , and G. H. Chong . 2015. “Textural and Rheological Properties of Stevia Ice Cream.” International Food Research Journal 22: 1544–1549.

[fsn372030-bib-0050] Puertollano, E. , S. Kolida , and P. Yaqoob . 2014. “Biological Significance of Short‐Chain Fatty Acid Metabolism by the Intestinal Microbiome.” Current Opinion in Clinical Nutrition and Metabolic Care 17: 139–144. 24389673 10.1097/MCO.0000000000000025

[fsn372030-bib-0051] Pujari, R. , and G. Banerjee . 2021. “Impact of Prebiotics on Immune Response: From the Bench to the Clinic.” Immunology and Cell Biology 99: 255–273. 32996638 10.1111/imcb.12409

[fsn372030-bib-0052] Ricci, A. , M. Cirlini , L. Calani , et al. 2019. “In Vitro Metabolism of Elderberry Juice Polyphenols by Lactic Acid Bacteria.” Food Chemistry 276: 692–699. 30409649 10.1016/j.foodchem.2018.10.046

[fsn372030-bib-0053] Rinninella, E. , and L. Costantini . 2022. “Polyunsaturated Fatty Acids as Prebiotics: Innovation or Confirmation?” Food 11: 146. 10.3390/foods11020146 PMC8774454 35053879

[fsn372030-bib-0054] Saad, N. , C. Delattre , M. Urdaci , J. M. Schmitter , and P. Bressollier . 2013. “An Overview of the Last Advances in Probiotic and Prebiotic Field.” LWT 50: 1–16.

[fsn372030-bib-0055] Salem, M. M. F. , F. A. Fathi , and R. A. Awad . 2005. “Production of Probiotic Ice Cream.” Polish Journal Of Food And Nutrition Sciences 55: 267–271.

[fsn372030-bib-0056] Selle, K. , and T. R. Klaenhammer . 2013. “Genomic and Phenotypic Evidence for Probiotic Influences of *Lactobacillus gasseri* on Human Health.” FEMS Microbiology Reviews 37: 915–935. 23488471 10.1111/1574-6976.12021

[fsn372030-bib-0057] Shokryazdan, P. , M. Faseleh Jahromi , B. Navidshad , and J. B. Liang . 2017. “Effects of Prebiotics on Immune System and Cytokine Expression.” Medical Microbiology and Immunology 206: 1–9. 27704207 10.1007/s00430-016-0481-y

[fsn372030-bib-0058] Silveira, R. V. , L. M. Li , and G. Castellano . 2023. “Texture‐Based Brain Networks for Characterization of Healthy Subjects From MRI.” Scientific Reports 13: 16421. 37775531 10.1038/s41598-023-43544-6 PMC10541866

[fsn372030-bib-0059] Sims, I. M. , J. L. Ryan , and S. H. Kim . 2014. “In Vitro Fermentation of Prebiotic Oligosaccharides by *Bifidobacterium lactis* HN019 and *Lactobacillus* spp.” Anaerobe 25: 11–17. 24239979 10.1016/j.anaerobe.2013.11.001

[fsn372030-bib-0060] Sonnenburg, E. D. , and J. L. Sonnenburg . 2014. “Starving Our Microbial Self: The Deleterious Consequences of a Diet Deficient in Microbiota‐Accessible Carbohydrates.” Cell Metabolism 20: 779–786. 25156449 10.1016/j.cmet.2014.07.003 PMC4896489

[fsn372030-bib-0061] Sorrenti, V. , S. Ali , L. Mancin , S. Davinelli , A. Paoli , and G. Scapagnini . 2020. “Cocoa Polyphenols and Gut Microbiota Interplay: Bioavailability, Prebiotic Effect and Impact on Human Health.” Nutrients 12: 1908. 32605083 10.3390/nu12071908 PMC7400387

[fsn372030-bib-0062] Tamime, A. Y. , M. Saarela , A. K. Sondergaard , V. V. Mistry , and N. P. Shah . 2005. “Production and Maintenance of Viability of Probiotic Microorganisms in Dairy Products.” In Probiotic Dairy Products, edited by A. Y. Tamime , 39–72. Blackwell.

[fsn372030-bib-0063] Tan, J. , C. McKenzie , M. Potamitis , A. N. Thorburn , C. R. Mackay , and L. Macia . 2014. “The Role of Short‐Chain Fatty Acids in Health and Disease.” Advances in Immunology 121: 91–119. 24388214 10.1016/B978-0-12-800100-4.00003-9

[fsn372030-bib-0064] Terefe, N. S. , and M. A. Augustin . 2019. “Fermentation for Tailoring the Technological and Health‐Related Functionality of Food Products.” Critical Reviews in Food Science and Nutrition 60: 2887–2913. 31583891 10.1080/10408398.2019.1666250

[fsn372030-bib-0065] Torrico, D. D. , J. Tam , S. Fuentes , C. Gonzalez‐Viejo , and F. R. Dunshea . 2020. “Consumer Rejection Threshold, Acceptability Rates, Physicochemical Properties, and Shelf‐Life of Strawberry‐Flavored Yogurts With Reductions of Sugar.” Journal of the Science of Food and Agriculture 100: 3024–3035. 32056214 10.1002/jsfa.10333

[fsn372030-bib-0066] Tripathi, M. K. , and S. K. Giri . 2014. “Probiotic Functional Foods: Survival of Probiotics During Processing and Storage.” Journal of Functional Foods 9: 225–241.

[fsn372030-bib-0067] Vénica, C. I. , M. C. Perotti , and C. V. Bergamini . 2014. “Organic Acids Profiles in Lactose‐Hydrolyzed Yogurt With Different Matrix Composition.” Dairy Science & Technology 94: 561–580.

[fsn372030-bib-0068] Ventura, M. , C. Canchaya , Z. Zhang , G. F. Fitzgerald , and D. van Sinderen . 2007. “Molecular Characterization of *hsp20*, Encoding a Small Heat Shock Protein of *Bifidobacterium breve* UCC2003.” Applied and Environmental Microbiology 73: 4695–4703. 17513584 10.1128/AEM.02496-06 PMC1932816

[fsn372030-bib-0069] Verduci, E. , E. Di Profio , A. Corsello , et al. 2021. “Which Milk During the Second Year of Life: A Personalized Choice for a Healthy Future.” Nutrients 13: 3412. 34684413 10.3390/nu13103412 PMC8540900

[fsn372030-bib-0070] Walait, M. , H. R. Mir , Z. Hassan , and J. I. Wattoo . 2022. “Cracking the Metabolic Engineering of Bacteria: Review of Methods Involved in Organic Acid Production.” Natural Resources for Human Health 2: 121–128.

[fsn372030-bib-0071] Wickner, S. , M. R. Maurizi , and S. Gottesman . 1999. “Posttranslational Quality Control: Folding, Refolding, and Degrading Proteins.” Science 286: 1888–1893. 10583944 10.1126/science.286.5446.1888

[fsn372030-bib-0072] Zhao, Y. S. , A. S. Eweys , J. Y. Zhang , et al. 2021. “Fermentation Affects the Antioxidant Activity of Plant‐Based Food Material Through the Release and Production of Bioactive Components.” Antioxidants 10: 2004. 34943107 10.3390/antiox10122004 PMC8698425

